# Off-label Use of the Optilume Drug-coated Balloon in the Treatment of Bladder Neck Stenosis and Vesicourethral Anastomosis Stenosis

**DOI:** 10.1016/j.euros.2025.03.002

**Published:** 2025-04-11

**Authors:** Georgi Tosev, Ivan Damgov, Franklin Kuehhas, Hendrik Borgmann, Julian Struck, Johannes Salem, Timur H. Kuru

**Affiliations:** a Mannheim Urology, Mannheim, Germany; b Department of Urology, University Hospital Heidelberg, Heidelberg, Germany; c Division of Pediatric Nephrology, Center for Pediatric and Adolescent Medicine, University of Heidelberg, Heidelberg, Germany; d Kuehhas Andrology, Vienna, Austria; e Department of Urology, Brandenburg Medical School Theodor Fontane, Brandenburg an der Havel, Germany

**Keywords:** Bladder neck stenosis, Vesicourethral anastomotsis stenosis, Optilume drug-coated balloon, Prostatic neoplasms, Prostatectomy, Voiding dysfunction, Benign prostatic hyperplasia

## Abstract

Bladder neck stenosis (BNS) and vesicourethral anastomosis stenosis (VUAS) are uncommon but clinically significant complications following transurethral surgery and radical prostatectomy (RP), often presenting important clinical challenges because of high recurrence rates with standard treatments. We retrospectively evaluated the efficacy of the Optilume paclitaxel-coated balloon in managing recurrent BNS and VUAS. Sixteen patients who had undergone open or robotic RP, transurethral resection of the prostate, or GreenLight laser photoselective vaporization of the prostate were assessed. At 12 mo after BNS/VUAS surgery, the International Prostate Symptom Score (IPSS), postvoid residual volume (PVR), and freedom from repeat intervention were evaluated. All patients remained free from repeat intervention and anatomic recurrence at 1-yr follow-up. Among the 16 patients (seven with preexisting incontinence), 12-mo follow-up demonstrated stable continence with no worsening, and all patients reported high satisfaction and willingness to repeat the procedure. Significant improvements in scores were observed for IPSS (*p* < 0.001; *n* = 16) and IPSS quality of life (*t* = 4.75, *p* < 0.001; *n* = 16), while PVR did not change significantly (*p* = 0.442; *n* = 8). These findings suggest that the Optilume paclitaxel-coated balloon is an effective off-label treatment for recurrent BNS and VUAS as applied in two urology practices in Germany, corroborating evidence from randomized controlled trials on the treatment of anterior urethral strictures.

**Patient summary:**

We evaluated off-label use of the new drug-coated Optilume balloon for treatment of recurrent narrowing of the bladder neck or VA (vesicourethral anastomosis; surgical join between the bladder and urethra) in patients who had previously undergone prostate surgery. Our findings show that this treatment significantly improved urinary function assessed 1 year after the procedure and prevented the need for further surgeries.

Bladder neck stenosis (BNS) and vesicourethral anastomosis stenosis (VUAS) [1] are significant complications that can occur following transurethral surgery or radical prostatectomy (RP). Although relatively uncommon, these conditions present substantial challenges because of their impact on urinary function and high recurrence rates. The incidence of BNS after transurethral resection of the prostate (TURP) ranges from 0.3% to 9.7%, while VUAS occurs in 1.4–9% of patients following RP [[2], [3], [4]]. Despite low incidence, the global volume of prostate surgeries means many patients are affected, highlighting the need for effective management.

Traditional treatments typically involve endoscopic incision [5,6] or transurethral resection of scar tissue, but these methods are associated with high recurrence rates and a higher risk of complications such as incontinence [7]. Recurrence rates remain high, with 27–30% of patients experiencing relapse after the initial intervention [8]. Complex urethral reconstruction [9,10] is often considered the next step, yet it too has significant recurrence rates, ranging from 35% [9] to 45.5% [10]. Consequently, new, minimally invasive treatments are critically needed to reduce BNS/VUAS recurrence, enhance recovery, and improve outcomes.

The Optilume drug-coated balloon (DCB; paclitaxel coating) is a novel approach for the treatment of recurrent bulbar strictures [11] and benign prostatic hyperplasia (BPH) [12]. This technology combines urethral dilation with delivery of paclitaxel, an antiproliferative agent that inhibits fibroblast growth and thereby reduces stricture recurrence [11]. Furthermore, this approach helps in maintaining luminal patency of the prostatic urethra following dilation [12].

In this retrospective study we evaluated the efficacy of the Optilume DCB in patients with BNS or VUAS, conditions that significantly impair urinary function after prostate surgery. Given the limited treatment options for these complications, we assessed off-label use of the Optilume DCB as a potential therapeutic strategy in this patient population.

Our retrospective case series included 16 consecutive patients treated between January 2021 and January 2023 with Optilume DCB dilation for BNS or VUAS. These patients had previously undergone TURP, GreenLight laser prostate ablation, or RP. The study included patients with confirmed BNS or VUAS, with concomitant urethral stricture in some cases. All patients gave written consent to the procedure after being informed about the off-label nature of the surgery and alternative standard therapeutic options. All patients refused standard therapeutic options. The inclusion criteria were male patients aged ≥18 yr who had undergone RP (VUAS case) or surgery for BPH (laser enucleation of the prostate or TURP; BNS cases). The study protocol was approved by the institutional ethics committee (approval number F-2023-048).

The procedure was conducted using a standardized technique performed by experienced surgeons (>500 Optilume procedures) at two large urological practices in Germany. Procedures were performed under sedation with Propofol or local anesthetic with intravenous Novalgin (2.5 g), and local anesthetic for the urethra. None of the patients required repeat treatment during the follow-up period.

A rigid cystoscope (20F diameter; Karl Storz or Richard Wolf) with a 0° telescope was used for all cystoscopic procedures. Antibiotic prophylaxis commenced 48 h before surgery and continued for 72 h postoperatively. Baseline urethrograms were performed to assess stenosis, followed by cystoscopy and placement of a 150-cm guidewire (Radifocus Terumo). A 24F uncoated balloon (UroMax) was introduced through the working channel of the cystoscope and predilated the BNS or VUAS lumen by ∼50%. Then a drug-coated balloon of 5 cm in length and 30F in diameter was inflated to 10 atmospheres for a minimum of 7 min. An image of the Optilume DCB device with its mechanism of action is shown is Fig. 1. A 14F bladder catheter was placed postoperatively for 48 h.

**Fig. 1:**
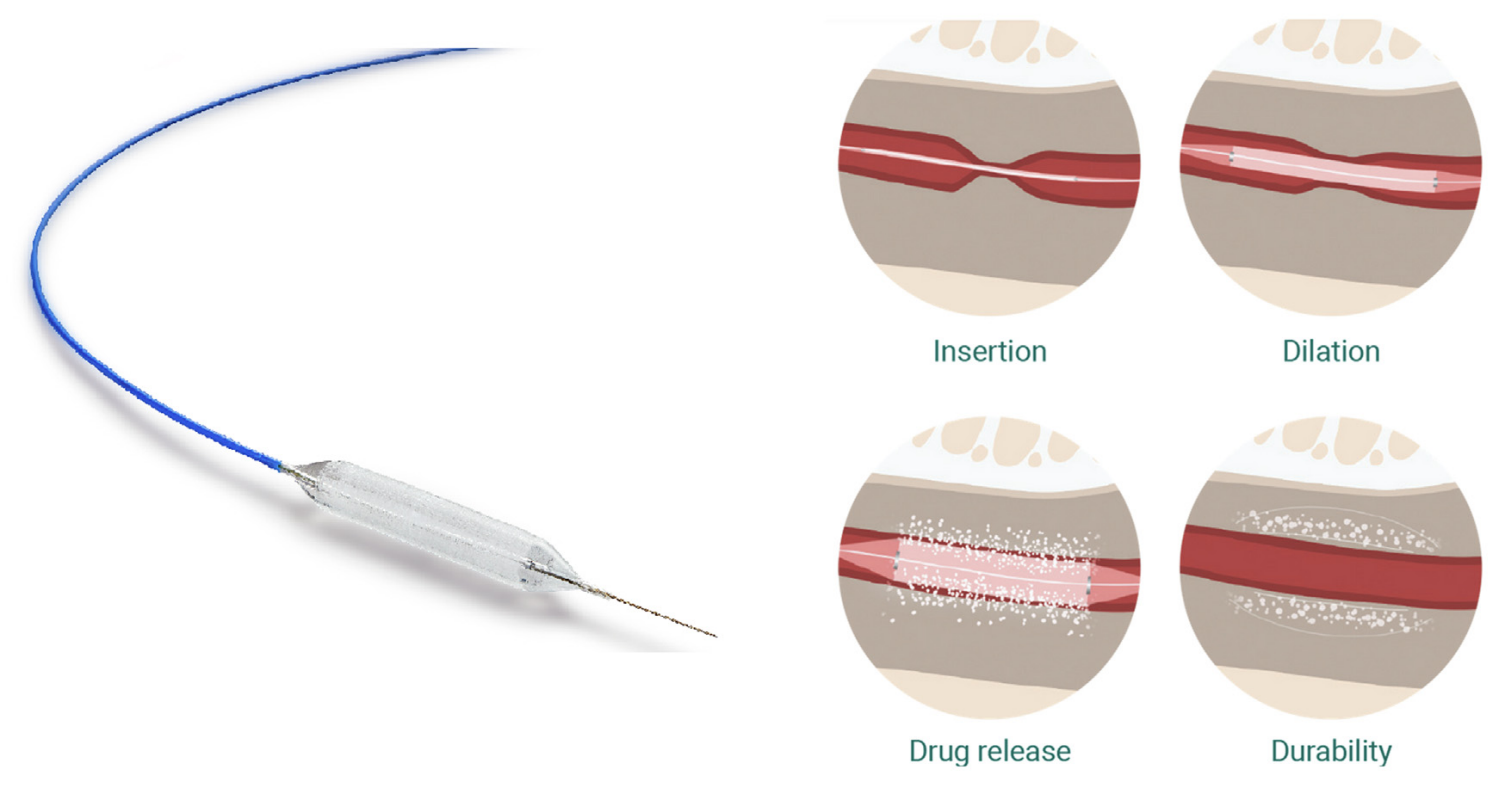
Left: the Optilume paclitaxel-coated balloon device featuring a specialized balloon catheter with a blue guidewire and drug-coated surface. Right: sequential illustration of the four key therapeutic phases. (1) Insertion: initial placement of the deflated balloon in the strictured area. (2) Dilation: mechanical expansion of the strictured segment. (3) Drug release: transfer of paclitaxel coating into the surrounding tissue, represented by white particles dispersing into the urethral wall. (4) Durability: maintenance of luminal patency following treatment, with a sustained drug effect in tissue. The cross-sectional views demonstrate the progressive treatment stages from stricture dilation to a sustained therapeutic response. Images provided by Laborie Germany and posted with their permission.

The primary outcome measure was the success rate, defined as the proportion of patients without anatomic recurrence or repeat intervention within the 1-yr follow-up period. Anatomic recurrence was defined as the reappearance of BNS or VUAS on follow-up imaging or endoscopic evaluation, characterized by narrowing at the surgical site that resulted in clinical symptoms or required repeat intervention. Secondary outcome measures included changes in the International Prostate Symptom Score (IPSS), IPSS quality of life (QoL) score, and postvoid residual volume (PVR), and an incontinence questionnaire asked at the 12-mo follow-up visit. Success rates were analyzed with stratification according to the number of prior endoscopic treatments; the impact of previous radiotherapy was also assessed. Descriptive statistics were used to summarize baseline characteristics. Paired *t* tests compared IPSS, IPSS QoL, and PVR before and 12 mo after surgery. Statistical significance was set at *p* < 0.05. The effect size for changes in IPSS and IPSS QoL was calculated using Cohen’s d.

Our study results are promising. All 16 patients remained free from repeat intervention and anatomic recurrence at 1-yr follow-up. The average patient age was 67.9 yr (range 56.8– 77.6 yr; Table 1). As summarized in Table 2, significant improvements were observed in IPSS, with a decrease in mean score from 28.1 at baseline to 14.6 at 12 mo after the intervention (*p* < 0.001), and IPSS QoL, with a decreased in mean score from 4.9 to 2.3 (*p* < 0.001). These changes corresponded to large effect sizes (Cohen’s d = 1.06 for IPSS and 1.19 for QoL). However, there was no significant change in PVR (*t* = 0.82, *p* = 0.442; Table 2), probably because of the small sample size for this analysis (*n* = 8); PVR could not be measured for the remaining eight patients at baseline as they were catheterized.

**Table 1:** Demographics and urological medical history for the 16 patients

Parameter	Result
Age (yr)	
Mean (standard deviation)	67.9 (5.74)
Median (range)	67.6 (56.8–77.6)
Male, *n* (%)	16 (100)
Suprapubic catheter at baseline, *n* (%)	1 (6.2)
Foley catheter at baseline, *n* (%)	6 (37.5)
Iatrogenic stricture etiology, *n* (%)	16 (100)
Type of surgery, *n* (%)	
Transurethral resection of the prostate	8 (50.0)
Radical prostatectomy	7 (43.8)
GreenLight laser	1 (6.2)
Number of previous endoscopic treatments, *n* (%)	
1	9 (56.2)
2	2 (12.5)
3	1 (6.2)
4	2 (12.5)
15	1 (6.2)
One previous robotic treatment, *n* (%)	2 (12.5)
Pelvic floor radiation, *n* (%)	7 (43.8)
Concomitant stricture, *n* (%)	
Bulbar stricture	4 (25.0)
Penile stricture	1 (6.2)
Undefined stricture	1 (6.2)
None	10 (62.5)

**Table 2:** Summary of results for IPSS, IPSS QoL, and PVR at baseline and 12 mo, with paired *t* tests

Parameter	Baseline	12 mo	Paired *t* test		
			*t* statistic	*p* value	Cohen’s *d*
**IPSS**					
Patients	16	16			
Mean (SD)	28.1 (6.90)	14.6 (8.08)	4.23	<0.001	1.06
Median (range)	30.5 (15–35)	13.5 (5–35)			
**IPSS QoL**					
Patients	16	16			
Mean (SD)	4.9 (1.09)	2.3 (1.54)	4.75	<0.001	1.19
Median (range)	5 (2–6)	2.0 (0–6)			
**PVR (ml)**					
Patients	8	8			
Mean (SD)	83.4 (92.86)	53.8 (45.26)	0.82	0.442	0.29
Median (range)	55 (10–300)	50 (10–150)			

IPSS = International Prostate Symptom Score; PVR = postvoid residual urine volume; QoL = quality of life.; SD = standard deviation.

In our cohort of 16 patients treated with the Optilume DCB, nine patients were continent before the procedure, while seven had preexisting urinary incontinence. Among those with incontinence, five had undergone RP, of whom four had received radiotherapy. The remaining two patients were in the TURP group; notably, one patient with a history of high-intensity focused ultrasound experienced grade I incontinence (requiring one pad/d), and the other reported grade II incontinence (from two to four pads/d) after multiple treatments over a 10-yr period. At 12-mo follow-up, the seven patients with baseline incontinence reported stable symptoms, and all 16 patients confirmed that their urinary incontinence had not worsened (Table 3). Furthermore, every patient stated that they would recommend the Optilume procedure to others with a similar diagnosis and would choose to undergo the procedure again if necessary (Table 3). Overall, these findings suggest that the Optilume procedure effectively maintained baseline continence levels in patients with preexisting incontinence and was associated with high patient satisfaction.

**Table 3:** Incontinence questions asked at 12-mo follow-up

Question	Response
How has your urinary incontinence been 12 months after the Optilume® procedure?	All 16 patients confirmed that their UI had not worsened. The seven patients with pre-existing UI reported that their level of UI was unchanged (stable) after the procedure
Do you think your urinary incontinence has worsened after the Optilume® procedure?	All 16 patients answered “No”
Would you recommend the Optilume® procedure to another patient with your diagnosis?	All 16 patients answered “Yes”
Would you undergo the Optilume® procedure again if you experience a recurrence of your previous diagnosis?	All 16 patients answered “Yes”

UI = urinary incontinence.

This retrospective analysis of patients treated with the Optilume DCB in two urology practices in Germany demonstrates for the first time that this strategy is a solution for the management of recurrent BNS and VUAS. The ability of the Optilume DCB to improve urinary function and prevent anatomic recurrence, even after multiple prior endoscopic treatments, is promising and underscores the short-term efficacy of the procedure. This is especially relevant given the high recurrence rates typically associated with traditional treatment methods [10,[12], [13], [14]] and builds on evidence from a recent case report for a single patient treated for prostate bladder neck stenosis [13]. The Optilume DCB delivers paclitaxel directly to the pathological area, inhibits fibroblast growth, and prevents the formation of scar tissue [14,15]. The minimally invasive nature of the procedure, which often negates the need for inpatient care, offers additional benefits in terms of patient comfort and health care cost savings.

In comparison to traditional treatment options, the Optilume DCB provides both mechanical dilation and localized drug delivery, and potentially offers longer-lasting results, as indicated by Robust Studies I and III. Traditional dilation combined with holmium laser incision has shown promising outcomes, with a recurrence rate of 2.44% after 12 mo [16]. By contrast, standard endoscopic treatments such as bladder neck incision (BNI) and transurethral resection are associated with significantly higher recurrence rates, ranging from 17.9% at 1 yr after transurethral incision [17] to 55% and 59.8% for transurethral resection in VUAS and BNS, respectively [18,19]. Furthermore, the rate of stricture recurrence and stone formation after use of the thermoxpandable Memokath 045 bladder neck stent for BNS is up to 7% at 12 mo [20]. Unlike the Optilume DCB, the Memokath 045 device does not deliver medication and may require periodic removal or replacement, with potential to increase the risk of complications.

Despite promising initial findings, our study has notable limitations. Its retrospective design, small sample size, and relatively short follow-up period may limit the generalizability of the results. Furthermore, the cost of the Optilume DCB, which is currently ∼€2600 in Germany, warrants consideration. Although this represents a substantial upfront investment, the potential reduction in retreatment rates, shorter procedural times, and quicker recovery may offset these expenses. A formal cost-effectiveness analysis in future studies could provide valuable insights, especially in health care systems with budgetary constraints. Larger, prospective studies with extended follow-up are needed to validate the efficacy of the Optilume DCB for BNS and VUAS and to explore its long-term risks and benefits.

Another key consideration is the off-label use of the Optilume device. Although off-label applications are recognized in clinical practice, they require careful ethical scrutiny. In this study, patients were informed about the off-label nature of the procedure, including its risks and benefits, and institutional review board approval was obtained. Such use can be advantageous when existing treatments are insufficient, and allows exploration of alternative therapeutic options. Nonetheless, patient safety remains the highest priority, and further research is necessary to establish robust evidence guiding both on-label and off-label use of this device. Given the small number of participants and the separate reporting for both BNS and VUAS cohorts, we chose to include both scenarios to reflect real-world clinical practices for conditions that often require timely intervention.

In conclusion, our study provides encouraging preliminary evidence regarding the efficacy of the Optilume DCB in managing recurrent BNS and VUAS. While these results are promising, further research, including larger-scale prospective trials and comprehensive cost-effectiveness analyses, is needed to confirm these findings and to better define the long-term potential of this innovative therapeutic approach.

  ***Author contributions***: Georgi Tosev had full access to all the data in the study and takes responsibility for the integrity of the data and the accuracy of the data analysis.

  *Study concept and design*: Tosev, Salem, Kuru.

*Acquisition of data*: Tosev, Salem, Kuru.

*Analysis and interpretation of data*: Tosev, Damgov, Kuehhas, Borgmann, Struck.

*Drafting of the manuscript*: Tosev, Damgov.

*Critical revision of the manuscript for important intellectual content*: Kuehhas, Borgmann, Struck.

*Statistical analysis*: Damgov.

*Obtaining funding*: None.

*Administrative, technical, or material support*: Borgmann, Struck, Salem, Kuru.

*Supervision*: Salem, Kuru.

*Other*: None.

  ***Financial disclosures:*** Georgi Tosev certifies that all conflicts of interest, including specific financial interests and relationships and affiliations relevant to the subject matter or materials discussed in the manuscript (eg, employment/affiliation, grants or funding, consultancies, honoraria, stock ownership or options, expert testimony, royalties, or patents filed, received, or pending), are the following: None.

  ***Funding/Support and role of the sponsor*:** None.

  ***Acknowledgments*:** We would like to sincerely thank Mr. Erwin Van Uffel for his suggestions on data analysis during the preparation of this manuscript, which were deeply appreciated.
